# Letter from the Editor in Chief

**DOI:** 10.19102/icrm.2018.090406

**Published:** 2018-04-15

**Authors:** Moussa Mansour


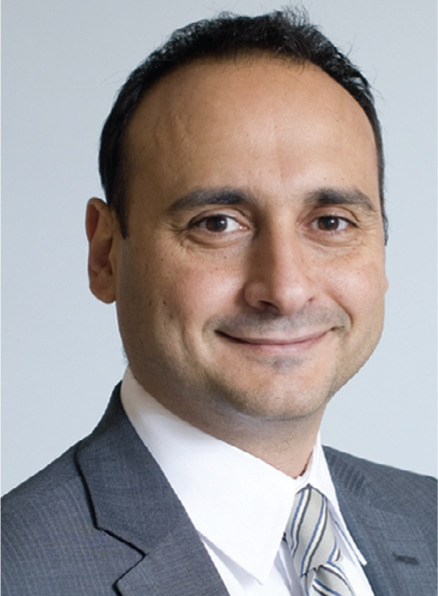


Dear Readers,

In the last several years, atrial fibrillation (AF) has become an epidemic in the United States. It is expected that, by the year 2050, between 12 and 15 million people in the country will have AF.^1^ This is expected to create a huge clinical and economical burden, mostly because of the stroke risk associated with this arrhythmia. Patients with AF are five times more likely to experience a stroke in comparison with those without. Oral anticoagulation is effective in reducing the rate of stroke secondary to AF. However, despite significant advances in the field of pharmacological therapy, only 40% of patients with AF who are at risk of stroke are taking oral anticoagulation.^2^

This issue of *The Journal of Innovation in Cardiac Rhythm Management* contains several articles on stroke prevention in patients with AF. I would like to highlight the article by Chava et al.^3^ reviewing the most recent advances in the field of left atrial appendage (LAA) closure. The authors describe US Food and Drug Administration-approved and investigational LAA closure devices including catheter-based and surgical technologies. They also summarize the findings of landmark clinical trials such as PROTECT AF^4^ and PREVAIL.^5^

This article is important in that it highlights the significance of the field of LAA closure. Historically, this field has been criticized for a lack of robust data. However, this is rapidly changing. Some of the current trials aim to test the efficacy and safety of new LAA closure tools and technological designs, such as the AMPLATZER™ Amulet™ LAA Occluder Trial (Amulet IDE) (NCT02879448); the Coherex WAVECREST I Left Atrial Appendage Occlusion Study (NCT02239887); and the Investigational Device Evaluation of the WATCHMAN FLX™ LAA Closure Technology (PINNACLE FLX) (NCT02702271) study. Others, such as the Assessment of the WATCHMAN™ Device in Patients Unsuitable for Oral Anticoagulation (ASAP-TOO) (NCT02928497) study, seek to identify new indications for this therapy. As a result, I believe that the next five years will be very exciting for this field.

I hope that you enjoy reading this issue of *The Journal of Innovations in Cardiac Rhythm Management* and that you find the content educational.

Sincerely,


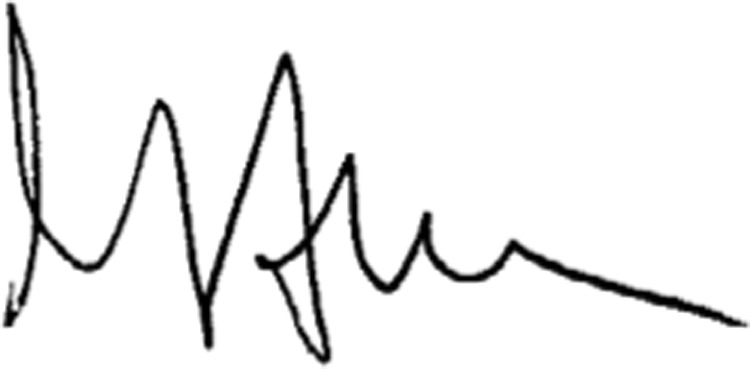


Moussa Mansour, MD, FHRS, FACC

Editor-in-Chief

The Journal of Innovations in Cardiac Rhythm Management

MMansour@InnovationsInCRM.com

Director, Atrial Fibrillation Program

Jeremy Ruskin and Dan Starks Endowed Chair in Cardiology

Massachusetts General Hospital

Boston, MA 02114

## References

[1] Miyasaka Y, Barnes ME, Gersh BJ (2006). Secular trends in incidence of atrial fibrillation in Olmsted County, Minnesota, 1980 to 2000, and implications on the projections for future prevalence.. Circulation..

[2] Marzec LN, Wang J, Shah ND (2017). Influence of direct oral anticoagulants on rates of oral anticoagulation for atrial fibrillation.. J Am Coll Cardiol..

[3] Chava R, Turagam MK, Lakkireddy D (2018). Left atrial appendage occlusion: what are the options and where is the evidence?. J Innov Cardiac Rhythm Manage..

[4] Reddy VY, Holmes D, Doshi SK, Neuzil P, Kar S (2011). Safety of percutaneous left atrial appendage closure: results from the Watchman Left Atrial Appendage System for Embolic Protection in Patients with AF (PROTECT AF) clinical trial and the Continued Access Registry.. Circulation..

[5] Holmes DR, Kar SK, Price MJ (2014). Prospective randomized evaluation of the Watchman Left Atrial Appendage Closure device in patients with atrial fibrillation versus longterm warfarin therapy: the PREVAIL trial.. J Am Coll Cardiol..

